# Omega-3 (*n*-3) Fatty Acid–Statin Interaction: Evidence for a Novel Therapeutic Strategy for Atherosclerotic Cardiovascular Disease

**DOI:** 10.3390/nu16070962

**Published:** 2024-03-27

**Authors:** Ivana Djuricic, Philip C. Calder

**Affiliations:** 1 Department of Bromatology, Faculty of Pharmacy, University of Belgrade, 11221 Belgrade, Serbia; ivana.djuricic@pharmacy.bg.ac.rs; 2 School of Human Development and Health, Faculty of Medicine, University of Southampton, Southampton SO16 6YD, UK; 3 NIHR Southampton Biomedical Research Centre, University Hospital Southampton NHS Foundation Trust and University of Southampton, Southampton SO16 6YD, UK

**Keywords:** *n*-3 fatty acid, statin, inflammation, SPM, lipid lowering, ASCVD

## Abstract

Managing atherosclerotic cardiovascular disease (ASCVD) often involves a combination of lifestyle modifications and medications aiming to decrease the risk of cardiovascular outcomes, such as myocardial infarction and stroke. The aim of this article is to discuss possible omega-3 (*n*-3) fatty acid–statin interactions in the prevention and treatment of ASCVD and to provide evidence to consider for clinical practice, highlighting novel insights in this field. Statins and *n*-3 fatty acids (eicosapentaenoic acid (EPA) and docosahexaenoic acid (DHA)) are commonly used to control cardiovascular risk factors in order to treat ASCVD. Statins are an important lipid-lowering therapy, primarily targeting low-density lipoprotein cholesterol (LDL-C) levels, while *n*-3 fatty acids address triglyceride (TG) concentrations. Both statins and *n*-3 fatty acids have pleiotropic actions which overlap, including improving endothelial function, modulation of inflammation, and stabilizing atherosclerotic plaques. Thus, both statins and *n*-3 fatty acids potentially mitigate the residual cardiovascular risk that remains beyond lipid lowering, such as persistent inflammation. EPA and DHA are both substrates for the synthesis of so-called specialized pro-resolving mediators (SPMs), a relatively recently recognized feature of their ability to combat inflammation. Interestingly, statins seem to have the ability to promote the production of some SPMs, suggesting a largely unrecognized interaction between statins and *n*-3 fatty acids with relevance to the control of inflammation. Although *n*-3 fatty acids are the major substrates for the production of SPMs, these signaling molecules may have additional therapeutic benefits beyond those provided by the precursor *n*-3 fatty acids themselves. In this article, we discuss the accumulating evidence that supports SPMs as a novel therapeutic tool and the possible statin–*n*-3 fatty acid interactions relevant to the prevention and treatment of ASCVD.

## 1. Introduction

Cardiovascular diseases (CVDs) remain the leading cause of mortality globally, being responsible for almost 18 million deaths each year [1]. CVDs are a group of disorders of the heart and blood vessels, and include coronary heart disease (CHD), peripheral vascular disease, cerebrovascular disease as well as other conditions. Mortality from CVDs is mainly due to myocardial infarction (heart attack) and stroke, which may occur prematurely in those aged under 70 years [1]. Management of risk factors remains a challenge in primary and secondary prevention of CVDs, with dyslipidemias being an important critical issue. Dyslipidemias reflect an imbalance of blood lipids and lipoproteins, characterized mainly by elevated plasma concentrations of low-density lipoprotein cholesterol (LDL-C) and triglyceride (TG) along with lowered concentrations of high-density lipoprotein cholesterol (HDL-C) [2]. The role of elevated LDL-C as a risk factor for atherosclerotic cardiovascular disease (ASCVD) is well recognized [3]. Furthermore, elevation in TGs and (TG)-rich lipoproteins (TGRLs) has also been recognized as an independent risk factor for CVDs, most likely due to the infiltration of TGRLs into the intimal space, initiating atherosclerotic plaque formation and promoting plaque progression [4]. It is estimated that globally nearly 4 million deaths per year are attributed to elevated LDL-C [5]. Furthermore, it is estimated that ASCVD causes about two thirds of all deaths from CVDs [5]. Familial or primary dyslipidemias are genetically determined, while secondary dyslipidemias are associated with other conditions, such as unhealthy behaviors, underlying diseases (e.g., obesity, diabetes mellitus), and applied drugs [6].

The most relevant and recent European and American guidelines suggest lipid-lowering treatment in those with increased risk of ASCVD [7]. For instance, the European Society of Cardiology guidelines recommend the use of statins, as well as some non-statin therapies, to reach LDL-C targets [8,9]. The 2019 American Heart Association/American College of Cardiology/MultiSociety guidelines have LDL-C thresholds and suggest combination therapies that are more stringent for selected patients [10,11]. Statins function by inhibiting the hydroxymethylglutaryl-CoA (HMG-CoA) reductase enzyme, so decreasing endogenous (mainly hepatic) cholesterol biosynthesis. As a result, hepatic expression of the LDL-receptor is upregulated, favoring hepatic clearance of LDL. The result of this is lowering the circulating concentrations of total cholesterol, LDL-C and, to a lesser extent, TGs. Statins have pleiotropic effects beyond lipid lowering [12]. These effects include improving endothelial function, inhibiting vascular inflammation and promoting plaque stability (see later sections).

There is overwhelming evidence that the omega-3 (*n*-3) polyunsaturated fatty acids (PUFAs) eicosapentaenoic acid (EPA) and docosahexaenoic acid (DHA) have a well-described TG-lowering effect [13]. The effect of these *n*-3 PUFAs on circulating TG concentrations is dose-dependent [14]. The decrease in circulating TG concentrations by *n*-3 PUFAs is likely due to a combination of factors, including an increase in the hepatic oxidation of fatty acids favoring their partitioning away from TG synthesis as well as other effects that reduce de novo fatty acid and TG synthesis and hepatic assembly of TGRLs [15,16]. *N*-3 PUFAs may also aid clearance of circulating TGs, for example, by increasing lipoprotein lipase activity [17]. Essentially, EPA and DHA influence the production of TGs, their release from the liver and their removal from the bloodstream, contributing to lower circulating levels [17,18]. Various international guidelines outline recommendations for using *n*-3 fatty acids to lower TG levels in individuals with elevated TGs; for example, the American Heart Association recommends up to 4 g/day EPA + DHA or EPA only to treat hypertriglyceridemia [13]. Just as for statins, *n*-3 PUFAs have a series of other cardioprotective benefits through different mechanisms [19], some of these overlapping the pleiotropic effects of statins [20].

Despite a large amount of clinical evidence that confirms the individual effects of statins and of *n*-3 PUFAs on blood lipid concentrations and on CVD outcomes, there is still a lack of consensus on how combined treatments might benefit these conditions. As inflammation is involved in various stages of ASCVD, anti-inflammatory actions of statins and of *n*-3 PUFAs are likely to be important. EPA and DHA are well described to exert anti-inflammatory actions, acting through several interacting mechanisms [21,22]. Furthermore, it is now known that both EPA and DHA are precursors for the biosynthesis of a family of lipid mediators (i.e., oxylipins), which together have been called specialized pro-resolving mediators (SPMs) [23]. There is also research suggesting that statins may indirectly affect anti-inflammatory/pro-resolving pathways; for example, statins might promote the production of specific SPMs (see later sections), suggesting an intriguing interaction between two lipid-lowering therapies to mitigate inflammation.

This article aims to discuss possible *n*-3 fatty acid–statin interactions in the prevention and treatment of ASCVD and to provide evidence to consider for clinical practice, highlighting novel insights in this field.

## 2. Biological Actions of Omega-3 (*n*-3) Fatty Acids and Their Oxylipin Metabolites

The human diet contains *n*-3 fatty acids from both plant and animal foods. Plants synthesize α-linolenic acid, and this fatty acid is an important constituent of many nuts, seeds, and plant oils. Plants do not produce EPA and DHA. These are mainly produced by algae and are passed through the aquatic food chain to fish. White (or lean) fish contain modest levels of EPA and DHA, typically up to 300 mg per adult serving [24], while oily (or fatty) fish contain high levels, typically between 1 and 2.5 g per adult serving [24]. Good sources of EPA and DHA include salmon, mackerel, herring, trout, and sardines. No foods other than seafood contain significant amounts of EPA and DHA unless they have been fortified, which is not common. EPA and DHA are also found in many “omega-3” supplements which include fish oils, krill oil, and algal oils [24]. The EPA and DHA content of supplements can vary, but a typical fish oil supplement usually contains about 300 mg of EPA + DHA per g of oil. Concentrates are available as supplements where EPA + DHA contribute as much as 60% of the oil. There are also highly concentrated pharmaceutical grade preparations which are 90% or more EPA + DHA or just EPA. Dietary intakes of EPA + DHA are low in those who do not regularly consume oily fish or use supplements containing EPA + DHA; such low intakes are estimated in the high tens to low hundreds of mg daily [25,26] and are generally below recommended intakes [27,28,29,30,31]. Regular consumption of oily fish or use of supplements, particularly concentrates, results in markedly higher intake of EPA and DHA and in higher blood, cell, and tissue levels of those two *n*-3 PUFAs [32,33]. The availability of EPA and DHA in supplements and in pharmaceutical-grade preparations has permitted the conduct of placebo-controlled randomized controlled trials (RCTs) of these *n*-3 PUFAs.

*n*-3 PUFAs, especially EPA and DHA, are known for their health benefits, particularly in relation to cardiovascular health [34]; these benefits come about through the favorable modulation of a broad range of well-described risk factors [19]. These include blood TG concentrations, systolic and diastolic blood pressure, thrombosis, endothelial function, inflammation, and cardiac function, which are all improved by *n*-3 PUFAs (see [19,34,35] and references therein). As already mentioned, EPA and DHA exert a number of actions that result in lower plasma TG levels [18]. These include the combination of increased hepatic fatty acid oxidation, reduced hepatic de novo lipogenesis and very-low-density lipoprotein (VLDL) synthesis [15,16], and increased clearance of TGRLs including chylomicrons and VLDLs, thus decreasing their half-life in the circulation [17,18]. Furthermore, EPA and DHA contribute to lower TG levels through decreased delivery of non-esterified fatty acids to the liver and increased hepatic synthesis of phospholipids rather than TGs [36]. Recent investigations suggest an additional novel mechanism by which EPA and DHA act to lower plasma TGs. With increased intake of EPA and DHA, EPA- and DHA-derived N-acyl taurines (NATs) accumulate in bile and plasma [37]. The DHA-containing NAT (C22:6 NAT) inhibits intestinal hydrolysis of TGs and lipid absorption, resulting in lower plasma TG levels and reduced risk of accumulation of TGs in the liver [37]. This might explain why DHA has a slightly greater impact on plasma TG concentrations than EPA does [38,39]. However, while both EPA and DHA show significant TG-lowering in humans, head-to-head studies report that they have an independent effect on different lipoprotein sub-particles; EPA lowers HDL3 while DHA increases HDL2, which is considered to be more cardioprotective [40,41]. Furthermore, DHA, but not EPA, can raise LDL-C to a small extent and increases LDL particle size [38,40,41]. Despite increasing LDL-C levels, DHA does not affect apolipoprotein B (Apo-B) concentration, which suggests the modification of LDLs to larger, less atherogenic particles [13,42]. On the other hand, EPA has distinct antioxidant benefits compared to DHA, which are suggested to be due to EPA’s optimal chain length and degree of unsaturation [43]. An in vitro study showed that EPA may orientate optimally within lipoprotein particles and membranes, inhibiting oxidation of LDL and altering membrane cholesterol domains, resulting in improved clearance of lipoproteins and reduced atherogenic activity [43]. Furthermore, biophysical evidence supports opposing effects of EPA and DHA on phospholipid interactions and cholesterol distribution within membranes [44,45]. EPA may maintain intermolecular phospholipid packing constraints and preserve the even distribution of cholesterol in membranes, while DHA induces membrane phospholipid disorder, which causes cholesterol to self-aggregate [45,46].

Low-grade inflammation has been long recognized to play a significant role in the formation, progression, and rupture of atherosclerotic plaques [47,48,49,50]. EPA and DHA modulate many relevant aspects of the inflammatory process, including the migration of leucocytes and the production of inflammatory chemokines, cytokines, and eicosanoids [21,22]. These anti-inflammatory effects involve the modulation of membrane events, cell signaling, transcription factor activation, and gene expression by EPA and DHA [22]. Further to this, it has now emerged that oxylipins produced from *n*-3 PUFAs are an important component of their bioactivity, including in the contexts of inflammation and CVDs.

Oxylipins are a class of bioactive lipid molecules generated by the oxidation of *n*-3 and *n*-6 PUFAs often in response to inflammatory stimuli [51,52,53]. They are produced through enzymatic and non-enzymatic oxidation pathways; enzymatic conversion of *n*-3 and *n*-6 PUFAs shares the same series of enzymes, i.e., lipoxygenases (LOXs), cyclooxygenases (COXs), and cytochromes P450 (CYP-450) [51,52,53]. Because different oxylipins have different biological actions, a balance among various oxylipins is required for normal physiological function, and dysregulation of their pathways of synthesis and/or action has been suggested to be involved in the pathogenesis of many diseases, including inflammatory disorders and CVDs [54]. Some oxylipins promote inflammation (e.g., many arachidonic acid (AA)-derived oxylipins such as several 2-series prostaglandins and 4-series leukotrienes [43]), but others have weaker pro-inflammatory, anti-inflammatory, and pro-resolving properties (e.g., oxylipins generated from EPA, docosapentaenoic acid (DPA), and DHA) [55].

**Figure 1:**
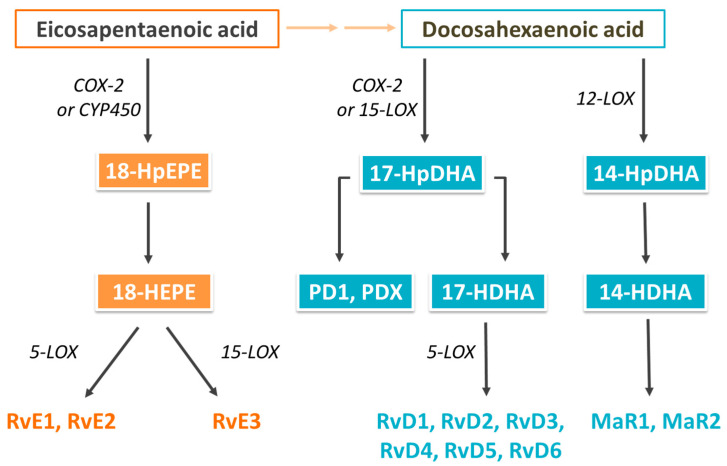
Overview of the pathways of synthesis of specialized pro-resolving mediators from EPA and DHA. Abbreviations used: COX, cyclooxygenase; CYP450, cytochrome P450; HDHA, hydroxy-DHA; HEPE, hydroxy-EPA; HpDHA, hydroperoxy-DHA; HpEPE, hydroperoxy-EPA; LOX, lipoxygenase; MaR, maresin; PD, protectin D; and Rv, resolvin.

Some oxylipins are termed SPMs since they act to resolve inflammation that is already established (e.g., by reducing production of pro-inflammatory cytokines) and promoting the clearance of inflammatory cells, debris, and pathogens [52,56,57]. SPMs are ligands for cell surface G-protein-coupled receptors (GPCRs), initiating pro-resolving responses in many inflammatory cell targets acting via various signaling pathways, such as mitogen-activated protein kinases, nuclear factor kappa B, phosphoinositide 3-kinase, and peroxisome proliferator-activated receptor γ [52,58,59]. Additionally, SPMs regulate specific microRNA signatures associated with the resolution of acute inflammation and apoptotic markers, such as B-cell lymphoma-2 and caspases 3 and 9 [60,61]. Examples of SPMs include maresins, resolvins, and protectins. SPMs synthesized from DHA include D-series resolvins (RvD), protectins, and maresins, while E-series resolvins (RvE) are synthesized from EPA [52,55,56]. These are produced through pathways that involve COX and LOX enzymes (Figure 1) [52,56,57,58].

The *n*-6 PUFA AA is a precursor for the synthesis of lipoxins (e.g., LXA4, LXB4, 15-epi-LXA4, 15-epi-LXB4) which are also SPMs; lipoxins are produced through 15-LOX and 5-LOX pathways and via transcellular biosynthesis pathways, including roles for 5-LOX and 12-LOX [62,63]. Lipoxins modulate the activity of immune cells, influencing their functions in a way that promotes resolution rather than sustained inflammation. This includes regulating the production of pro-inflammatory mediators [63].

## 3. Oxylipins in Cardiovascular Diseases

Chronic unresolved inflammation and continuous release of pro-inflammatory mediators is involved in the development and progression of atherosclerosis [47,48,49,50]. Recent studies on CVDs reported oxylipin production to be dysregulated [64,65,66]. In this regard, a cross-sectional study has reported novel insights into the oxylipin profile, finding an increased salivary maresin concentration and decreased protectin concentrations in patients with CVD compared to healthy controls [67]. The salivary levels of protectins and maresins were independent of other confounding factors contributing to CVDs [67]. Plasma levels of SPMs and pro-inflammatory oxylipins (i.e., leukotriene (LT) B4 and prostaglandins) were measured in patients with coronary artery disease (CAD) to explore the association with coronary plaque progression [68]. Higher plasma levels of the *n*-3 PUFAs EPA + DHA were associated with significantly higher plasma levels of resolvin E1 (RvE1) and its precursor 18-HEPE. Patients with low plasma EPA + DHA levels had low (18-HEPE + RvE1)/LTB4 ratios and significant plaque progression compared to those with high plasma EPA + DHA levels and high (18-HEPE + RvE1)/LTB4 ratios, who showed significant plaque regression [68]. It has also been reported that atherosclerotic plaque SPM levels are altered in CVD, with markedly lower concentrations of RvD1 in vulnerable, compared with non-vulnerable, regions of human carotid atherosclerotic plaques [69]. Furthermore, plasma levels of 15-epi-LXA4 were significantly lower in patients with symptomatic peripheral atherosclerosis than in healthy individuals [70]. Circulating concentrations of an *n*-3 DPA-derived resolvin (RvD*n*-3 DPA) were lower in patients with CVD than in healthy controls [71]. A decrease in RvD*n*-3 DPA concentrations was associated with increased blood vessel inflammation and progression of vascular disease [71]. Plasma SPMs were profiled in patients with ST-elevation myocardial infarction (STEMI) in the first week following myocardial infarction. Patients with STEMI had remarkably increased SPM levels compared to stable CAD patients and healthy controls, suggesting that protective pro-resolving mechanisms are activated after myocardial infarction [64]. The observed increase in SPMs mainly involved an increase in protectins formed from DPA and DHA. Furthermore, a shift in 5-LOX activity away from LTB4 to the pro-resolving *n*-3 DPA-derived RvTs was observed [64].

The above observations suggest that one mechanism of the protective and therapeutic actions of *n*-3 PUFAs in CVDs involves the generation and activity of SPMs. Further to this, SPMs themselves may be novel therapeutic agents in ASCVD, a notion supported by studies involving exogenous administration of synthetic SPM analogs [72]. Concerning this, in a rabbit model of atherosclerosis, topical application of RvE1 attenuated atherosclerotic plaque formation and lowered C-reactive protein (CRP) levels [73]. In a study in Apoe^−/−^ mice, the administration of RvD2 and MaR1 prevented the development of vascular lesions leading to atheroma formation [74]. Additionally, the SPMs induced a significant alteration in the phenotype of macrophages from an inflammatory to a reparative state [74]. Similarly, systemic administration of RvD2 and MaR1 attenuated intimal hyperplasia in mice [75]. In a carotid artery balloon injury model in rats, administration of RvD1 or PD1 decreased muscle cell proliferation and leukocyte infiltration [76]. RvE1 decreased atherosclerotic lesion size and diminished severe lesion development in mice [77].

## 4. The Pharmacology of Statins

Statins are commonly prescribed medications that lower blood total cholesterol and LDL-C levels. They act through inhibition of the enzyme hydroxymethyl-glutaryl coenzyme A (HMG-CoA) reductase, which catalyzes the rate-limiting reaction in the pathway of hepatic cholesterol biosynthesis [78]. By decreasing cholesterol synthesis, statins help to lower LDL-C, which is pro-atherogenic, and may also modestly increase HDL-C, which is anti-atherogenic. Beyond lowering plasma total cholesterol and LDL-C levels, statins are now known to exert pleiotropic effects [12], as reviewed elsewhere recently [79]. These include improving endothelial function and vascular tone, reducing inflammation and oxidative stress, modulating platelet reactivity, and increasing atherosclerotic plaque stability [79].

Currently, seven forms of statins are marketed and commonly used to treat hypercholesterolemia and CVD risk. They are classified as fully synthetic compounds (atorvastatin, rosuvastatin, fluvastatin, and pitavastatin) and naturally occurring statins originally discovered in fungi (lovastatin, pravastatin, and simvastatin). With respect to the ability to reduce LDL-C concentrations, statins may be further classified as “strong” statins (rosuvastatin, pitavastatin, atorvastatin) and “weak” statins (pravastatin, simvastatin) [79]. Additionally, the definition “intensity of statin therapy” refers to the use of statin medications at doses that substantially reduce cholesterol levels, specifically LDL-C (Table 1) [80]. High-intensity statin therapy involves using statins at higher doses in order to cause at least a 50% decrease in LDL-C levels. Moderate-intensity statin therapy involves using statins at doses that lead to a moderate reduction (30–50%) in LDL-C levels. In low-intensity statin therapy, the daily dose needed to cause < 30% lowering of LDL-C levels is used.

## 5. Interrelationship between Omega-3 (*n*-3) PUFAs and Statins in Cardiovascular Diseases: Involvement of SPMs

Interactions between *n*-3 PUFAs and statins in the context of risk factors for CVDs have been previously discussed [20]. Since the primary action of statins is to lower plasma LDL-C levels and a key action of *n*-3 PUFAs is to lower plasma TG levels, they have synergistic effects on dyslipidemia. Statins and DHA both raise HDL-C levels. Statins and *n*-3 PUFAs have other overlapping actions; for example, both enhance endothelial nitric oxide synthesis.

**Figure 2:**
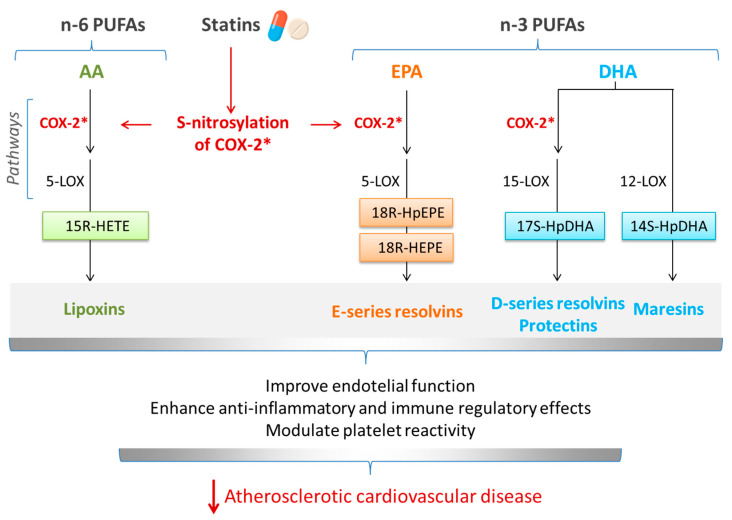
Statins can induce specialized pro-resolving mediator production via S-nitrosylation of cyclooxygenase-2 (COX-2) mitigating the inflammatory processes underlying atherosclerotic cardiovascular diseases. Abbreviations used: COX-2*, S-nitrosylated COX-2; *n*-6 PUFAs, *n*-6 polyunsaturated fatty acids; *n*-3 PUFAs, *n*-3 polyunsaturated fatty acids; AA, arachidonic acid; EPA, eicosapentaenoic acid, DHA, docosahexaenoic acid; 5-LOX, 5-lipoxygenase; 15-LOX, 15-lipoxygenase; 12-LOX, 12-lipoxygenase; 5R-HETE, 5R-hydroxy-eicosatetraenoic acid; 18R-HpEPE, 18R-hydroperoxy-eicosapentaenoic acid; 18R-HEPE, 18R-hydroxy-eicosapentaenoic acid; 17S-HpDHA, 17S-hydroperoxy-docosahexaenoic acid; 14S-HpDHA, 14S-hydroperoxy-docosahexaenoic acid.

Inflammation is another common target of statins and *n*-3 PUFAs. The anti-inflammatory and pro-resolving actions of *n*-3 PUFAs, and the involvement of EPA- and DHA-derived SPMs in these actions, were described earlier. Statins have been shown to reduce the levels of key markers of inflammation, such as CRP and interleukin-6 (IL-6) [81], and they influence the function of immune cells (e.g., macrophages and T lymphocytes) [82,83], which play essential roles in inflammation. By modulating the activity of these cells, statins can dampen the inflammatory response. Since inflammation plays a pivotal role in endothelial dysfunction and in atherosclerotic plaque instability, the effect of statins is to improve endothelial function and to stabilize atherosclerotic plaques, making them less prone to rupture and reducing the risk of cardiovascular events [84]. In addition to direct anti-inflammatory actions, statins can affect the expression and activity of key enzymes involved in the pathway of synthesis of oxylipins, resulting in the enhanced production of at least some SPMs (see [85] and references therein). One mechanism of action involves the S-nitrosylation of COX-2, which promotes the production of 15R-hydroxyeicosatetraenoic acid (15R-HETE) from AA, which further serves as a substrate for the 5-LOX-mediated conversion to 15-epi-lipoxins (Figure 2). Similarly, statins can induce the generation of resolvins and protectins from EPA and DHA, again as a result of S-nitrosylation of COX-2 (Figure 2).

Statins also regulate the levels of enzymes involved in oxylipin synthesis, and this has been studied in the context of CVDs. For example, in a rabbit model of atherosclerosis, atorvastatin treatment decreased expression of mRNA for 5-LOX-activating protein and the leukotriene receptor cysLT1R along with lowering serum LTD4 levels, all associated with stabilization, and even regression, of carotid atherosclerotic plaques [86]. Atorvastatin increased levels of LXA4 and 15-epi-LXA4 in the rat myocardium, which was linked with overexpression of 5-LOX and COX-2 in myocardial cells. A study with rats showed that atorvastatin at different doses markedly increased COX-2 and 15-epi-LXA4 levels in heart tissue [87]. A study on atorvastatin-treated rats also showed overproduction of 15-epi-LXA4 and increased COX-2 and 5-LOX levels in the cytosolic fraction of myocardial cells [88]. In diabetic mice, rosuvastatin increased 15-epi-LXA4 levels and decreased atherosclerotic plaque area, inflammation, and progression of atherosclerosis [89]. Thus, enhanced 15-epi-LXA4 production is a common finding in these studies of statins in laboratory animals; this could involve increased expression of the relevant synthetic enzymes (COX-2 and 5-LOX) and increased activity of COX-2 due to S-nitrosylation. As such, up-regulation of the lipoxin pathway could be a novel mechanism for statin-induced anti-inflammatory and immune regulatory effects. There may also be interaction of statins with *n*-3 PUFAs, in part through S-nitrosylation of COX-2 as described above, but there are other relevant observations. For example, statins resulted in higher expression of the RvE1 receptor (Erv1/ChemR23) in macrophages from the proximity of the necrotic core of atherosclerotic plaques [90]. The combination of RvE1 and atorvastatin has been tested in mice [77].

## 6. Omega-3 (*n*-3) Fatty Acids and Cardiovascular Diseases: Findings from Cohort Studies

Clues to the impact of dietary EPA + DHA on CVDs came from early studies in the native Inuit population of Greenland, who showed a much lower rate of mortality from myocardial infarction (MI) and from CHD than anticipated [91,92] with these effects being linked with the very high intake of EPA and DHA from their diet [93]. Observations of low risk of CVDs with high EPA and DHA intake were replicated in other populations from the Arctic [94] and Japan [95]. Multiple studies have reported inverse associations between dietary intake of EPA and DHA, or their levels in the blood or tissues, and risk of various CVD outcomes (see [96]). For example, data from the Nurse’s Health Study showed an inverse dose-dependent association of risk for developing CHD, having a non-fatal MI, or dying from CHD across quintiles of intake of EPA + DHA [97]. All three outcomes were decreased by about 50% in those with the highest intake compared with the lowest intake of EPA + DHA [97]. The prospective National Institutes of Health AARP Diet and Health Study included ~420,000 participants and had a 16-year follow-up; there was a significant inverse association between EPA + DHA intake and various mortality outcomes [98]. For example, EPA + DHA intake was associated with 15% and 18% lower CVD mortality in men and women, respectively, across extreme quintiles. Chowdhury et al. [99] reported that the combined data on dietary intake of EPA + DHA from 16 studies involving over 422,000 individuals demonstrated a 13% reduction in the risk of coronary outcomes for those in the top tertile of intake compared with those in the lower tertile. More recently, Alexander et al. [100] combined data from 17 cohort studies and found that the risk of CHD, fatal coronary events, coronary death, or sudden cardiac death was lower by 18%, 23%, 19%, and 47%, respectively, in those with higher dietary intake of EPA + DHA compared to those with lower intake.

Going beyond measurements of dietary intake of EPA and DHA, a number of studies have examined the associations between the concentrations of EPA + DHA in blood or blood pools (e.g., plasma, serum, serum lipids, or red blood cells) and CVD morbidity and mortality (see [96]). In the Physician’s Health Study, there was an inverse dose-dependent association of risk for sudden death across quartiles of whole blood EPA + DHA, with an 80% lower risk in those with the highest whole blood EPA + DHA concentrations compared to those with the lowest whole blood EPA + DHA concentrations [101]. Chowdhury et al. [99] combined data from 13 studies involving over 20,000 individuals in a meta-analysis that showed risk reductions for coronary outcomes of 22%, 21%, and 25% for those in the top tertile of circulating EPA, DHA and EPA + DHA, respectively, compared with those in the lower tertile. del Gobbo et al. [102] pooled data from 19 studies involving over 45,000 individuals; they found that each standard deviation increase in the EPA or DHA content of a blood or tissue pool was independently associated with a 10% lower risk of fatal CHD. More recently, data from 10 cohort studies identified a 15% reduction in the risk of fatal CHD with each one standard deviation increase in omega-3 index (EPA + DHA as a percentage of total fatty acids in red blood cells) [103]. Another pooling study of data from 17 prospective cohort studies with over 42,000 participants reported a lower risk of death from CVDs in those with the highest tertile of either EPA or DHA or EPA + DHA in a blood pool [104].

Taken together, this literature base provides consistent evidence that higher dietary intake of EPA and DHA, which results in higher blood and tissue levels of these two *n*-3 fatty acids, is associated with a lower risk of developing CHD and CVDs and of dying as a result of these diseases. Definitive proof of a cause-and-effect relationship can only come from RCTs.

## 7. Clinical Trials on Therapeutic Effects of Combined Statins and Omega-3 (*n*-3) Fatty Acids in Cardiovascular Diseases

A small number of large, long-term clinical trials have evaluated the effect of *n*-3 PUFAs combined with statins on cardiovascular outcomes in patients with high cardiovascular risk. The first of these studies was the important Japan EPA Lipid Intervention Study (JELIS), published in 2007. JELIS enrolled over 18,000 patients who had total cholesterol concentrations ≥ 6.5 mmol/L and LDL-C ≥ 4.4 mmol/L. These patients were randomized to receive either a statin alone or a statin along with highly concentrated EPA (1.8 g/d EPA as an ethyl ester) with a follow-up of 5 years [105]. Patients received 5 mg of simvastatin or 10 mg of pravastatin once daily as the first line of treatment. For uncontrolled hypercholesterolemia, the daily dose was increased to 10 mg of simvastatin or 20 mg of pravastatin. The combination of EPA-ethyl ester and statin had the same effect as statin alone on serum LDL-C levels in hypercholesterolemic patients: there was a 25% decrease in LDL-C concentrations in both groups. In the primary prevention arm of the trial, the primary outcome, which included a major coronary event (i.e., sudden cardiac death, fatal and non-fatal MI) or another non-fatal event (i.e., unstable angina pectoris, angioplasty, stenting, and coronary artery bypass grafting), was not significantly different between the groups receiving statins alone or statins plus EPA-ethyl ester. In the secondary prevention arm of the trial, which involved those patients with a history of CAD, non-fatal coronary events were significantly reduced by 19% in patients receiving statins plus EPA versus those receiving statins alone [105].

The Reduction of Cardiovascular Events with Icosapent Ethyl Intervention Trial (REDUCE-IT) [106] included 8179 patients with established CVD or with diabetes (58% of all patients had type 2 diabetes mellitus) and other risk factors. Of all participants, 62% received moderate-intensity statins, 31.5% high-intensity statins, and the rest low-intensity statins. Plasma LDL-C concentrations were well-controlled with statin therapy (baseline range was 1.06 to 2.59 mmol/L), but fasting TG levels were borderline and moderately elevated (1.52 to 5.63 mmol/L). Patients received either 4 g/day of EPA-ethyl ester (referred to as icosapent ethyl—the same concentrated pharmaceutical-grade preparation as used in JELIS), providing 3.6 g/day of EPA or a placebo (mineral oil). Median follow-up time was 4.9 years. The primary outcome (a composite of cardiovascular death, non-fatal stroke, non-fatal myocardial infarction, coronary revascularization, or unstable angina) was significantly improved in the icosapent ethyl group compared to the mineral oil control group (hazard ratio (HR): 0.75; 95% confidence interval (CI): 0.68, 0.83; *p* < 0.001). Icosapent ethyl also improved the main pre-specified secondary outcome (a composite of cardiovascular death, non-fatal myocardial infarction, or non-fatal stroke) (HR: 0.80; 95% CI: 0.66, 0.98; *p* = 0.03) as well as a whole range of other clinical outcomes [106]. Serum EPA increased 3.6-fold over the period of icosapent ethyl intervention, and the increase was associated with improvements in clinical endpoints [107].

Results from the EVAPORATE (Effect of Vascepa on Improving Coronary Atherosclerosis in People with High Triglycerides Taking Statin Therapy) trial suggest that EPA has benefits related to plaque reduction and stabilization in those taking statins [108]. EVAPORATE enrolled 80 patients with coronary atherosclerosis (one or more angiographic stenoses with ≥20% narrowing) and elevated fasting TG levels (1.5–5.6 mmol/L). All patients were receiving stable statin therapy. They had no history of myocardial infarction, stroke, or life-threatening arrhythmia within the previous six months. EVAPORATE used the same dose of icosapent ethyl as in REDUCE-IT. The EVAPORATE trial reported a significant regression of atherosclerotic plaques over 18 months in patients receiving icosapent ethyl [108].

Findings of STRENGTH (Long Term Outcomes Study to Assess Statin Residual Risk with Epanova in High Cardiovascular Risk Patients with Hypertriglyceridemia) [109] differ from those of REDUCE-IT, although the study design, target populations, and assessed primary outcomes were similar in both trials and both used “high-dose” *n*-3 PUFAs. In STRENGTH, patients with hypertriglyceridemia, high cardiovascular risk, and those receiving standard statin therapy received either 2.2 g EPA + 0.8 g DHA daily (as free fatty acids) or corn oil as a placebo. The different findings of REDUCE-IT and STRENGTH have been the subject of much discussion, especially given the apparently similar interventions and study designs. However, here are some differences that might be important. Firstly, REDUCE-IT used highly purified EPA (as an ethyl ester), while STRENGTH used both EPA and DHA (as free fatty acids). Secondly, REDUCE-IT used a higher overall dose of EPA (3.6 vs. 2.2 g/day) and did not use DHA. Thirdly, the two studies used different placebos (mineral oil vs. corn oil). Finally, REDUCE-IT had a higher proportion of secondary prevention patients with a high risk of cardiovascular events than STRENGTH.

There is an ongoing trial of relevance. This is the Evaluation in Secondary Prevention Efficacy of Combination Therapy-Statin and Eicosapentaenoic Acid (RESPECT-EPA) trial. RESPECT-EPA has enrolled 2460 patients with CAD who had received standard statin therapy for >1 month and who had a low plasma ratio of EPA to AA ratio (≤0.4). Patients were randomized to 1.8 g/day of highly purified EPA (as ethyl ester) or control. The primary endpoint of RESPECT-EPA is the composite of cardiovascular death, non-fatal myocardial infarction, non-fatal ischemic stroke, unstable angina pectoris, or clinically indicated coronary revascularization [110]. The selection of patients based on plasma EPA to AA ratio is a novelty for this type of trial, the rationale being that epidemiological studies report an inverse association between plasma ratio of EPA to AA and cardiovascular events and other atherosclerotic outcomes [111,112]. RESPECT-EPA has yet to report.

## 8. Meta-Analyses of Clinical Trials of Statins and *n*-3 PUFAs and Blood Lipids and Cardiovascular Diseases

AbuMweiss et al. published a comprehensive meta-analysis of RCTs of *n*-3 PUFAs (EPA + DHA) on cardiovascular risk factors, including blood lipids [19]. Using data from 110 RCTs, EPA + DHA were shown to markedly lower blood TG concentrations (effect size: −0.368; 95% CI: −0.427, −0.309; *p* = 0.0001) and to raise blood HDL-C concentrations (effect size: 0.039; 95% CI: 0.024, 0.054; *p* = 0.0001). LDL-C concentrations were also elevated (effect size: 0.150; 95% CI: 0.058, 0.243; *p* = 0.001), likely due to the aforementioned increase in the size of LDL particles, making them less atherogenic. This meta-analysis also reported significant benefits of EPA + DHA on other risk factors for CVDs, including systolic and diastolic blood pressure, heart rate, and the inflammatory biomarker CRP [19]. A meta-analysis of 16 RCTs of *n*-3 PUFAs (twelve of EPA + DHA, two of DHA, one of EPA, and one on α-linolenic acid) reported a significant improvement in vascular function assessed by flow-mediated dilatation [113]. Multiple meta-analyses of RCTs of EPA + DHA report reductions in blood concentrations of CRP, IL-6, and tumor necrosis factor [114,115,116,117,118]. These findings emphasize the broad-ranging beneficial actions of *n*-3 PUFAs, especially EPA and DHA, on risk factors for CVDs. There are also a number of meta-analyses of trials of *n*-3 PUFAs conducted in at-risk patients and reporting on clinical outcomes; meta-analyses published up to 2018, which are mostly favorable, are collated and summarized elsewhere [35]. In 2019, Hu et al. published a meta-analysis of 12 or 13 RCTs (depending upon the outcome) of EPA + DHA (or EPA alone) on clinical outcomes [118]. They identified that *n*-3 PUFAs decreased the risk of CVDs (rate ratio (RR): 0.95; 95% CI: 0.82, 0.98; *p* < 0.001), CVD death (RR: 0.92; 95% CI: 0.88, 0.97; *p* < 0.001), CHD (RR: 0.93; 95% CI: 0.89, 0.96; *p* < 0.001), CHD death (RR: 0.92; 95% CI: 0.86, 0.98; *p* = 0.014), myocardial infarction (RR: 0.88; 95% CI: 0.83, 0.94; *p* < 0.001), and major vascular events (RR: 0.95; 95% CI: 0.93, 0.98; *p* < 0.001). The meta-analysis of Bernasconi et al. [119], published in 2021, included a larger number of RCTs (14 to 39, depending upon the outcome). This meta-analysis identified that *n*-3 PUFAs decreased the risk of CVD events (relative risk (RelR): 0.95; 95% CI: 0.90, 1.00), CHD events (RelR: 0.90; 95% CI: 0.84, 0.97), CHD death (RelR: 0.91; 95% CI: 0.85, 0.98), myocardial infarction (RelR: 0.87; 95% CI: 0.80, 0.96), and fatal myocardial infarction (RelR: 0.65; 95% CI: 0.46, 0.91). Both Hu et al. [118] and Bernasconi et al. [119] included REDUCE-IT but not STRENGTH.

A small number of meta-analyses have attempted to decipher whether the combination of statins and *n*-3 PUFAs has a different effect on blood lipids compared to either therapy alone. A 2018 meta-analysis of six RCTs evaluated the effects of statin monotherapy versus combination therapy of statins and *n*-3 PUFAs on blood lipids [120]. The dosage of *n*-3 PUFAs used in the included trials was 2 or 4 g/day along with moderate-intensity statin therapy, and the duration of included trials was 6 to 16 weeks. There was no significant difference in LDL-C between the two groups, suggesting that *n*-3 PUFAs do not interfere with the LDL-C-lowering effect of statins, and that statins mitigate the LDL-C-raising effects of *n*-3 PUFAs. However, the combination therapy caused a greater decrease in the ratio of total cholesterol to HDL-C than statin monotherapy (standard mean difference (SMD): −0.215; 95% CI: −0.359, −0.071). Thus, this meta-analysis suggests that there is an advantage in the combination of satins plus *n*-3 PUFAs compared to statins alone [120].

A more recent meta-analysis of 32 RCTs involving 15,903 participants evaluated the effect of *n*-3 fatty acids or their combination with statins on the lipid profile in patients with hypertriglyceridemia [121]. The mean baseline TG level ranged from 154.2 to 699 mg/dL. Study duration varied from 4 to 48 weeks and dosage of EPA + DHA ranged from 1.24 to 4 g/day. The individual dosage of EPA and DHA was 0.6–4 g/day and 3–4 g/day, respectively. *N*-3 fatty acids as monotherapy decreased TGs (mean difference (MD): −39.81; 95% CI: −54.94, −24.69; *p* < 0.001), total cholesterol (MD: −2.98; 95% CI: −5.72, −0.25; *p* = 0.03), VLDL-C (MD: −25.12; 95% CI: −37.09, −13.14; *p* < 0.001), and non-HDL-C (MD: −5.42; 95% CI: −8.06, −2.78; *p* < 0.001) and increased LDL-C (MD: 9.10; 95% CI: 4.27, 13.94; *p* < 0.001) and HDL-C (MD: 1.60; 95% CI: 0.06, 3.15; *p* = 0.04). The cholesterol-lowering effect only occurred for combinations of EPA and DHA and at doses ≥ 4 g/day; DHA alone increased total cholesterol. There was no significant effect of *n*-3 PUFAs on apolipoprotein B (Apo-B) and apolipoprotein AI (Apo-AI) concentrations. The combination of *n*-3 fatty acids and statins significantly decreased TGs (MD: −29.63; 95% CI: −36.24, −23.02; *p* < 0.001), total cholesterol (MD: −6.87; 95% CI: −9.30, −4.45; *p* < 0.001), VLDL-C (MD: −20.13; 95% CI: −24.76, −15.50; *p* < 0.001), non-HDL-C (MD: −8.71; 95% CI: −11.45, −5.98; *p* < 0.001), Apo-B (MD: −3.50; 95% CI: −5.37, −1.64; *p* < 0.001), and Apo-AI (MD: −2.01, 95% CI: −3.07, −0.95; *p* < 0.001). The combination of *n*-3 PUFAs and statins caused a greater decrease in total cholesterol than *n*-3 PUFAs alone. However, the combined therapy did not significantly change the levels of HDL-C and LDL-C compared to the control group, a lack of effect that is difficult to explain and which contrasts with the findings of Choi et al. [120]. 

A meta-analysis and network meta-analysis published in 2020 reported the comparative efficacy of a statin vs. a statin, a statin vs. an *n*-3 PUFA, or a statin or *n*-3 PUFA vs. control for the prevention of total CVD, CHD, myocardial infarction, and stroke [122]. The analysis included 63 RCTs involving 264,516 patients; of these, 45 RCTs used statins, and 18 used *n*-3 PUFAs. Median follow-up was 3.7 years. Overall lipid-lowering therapy (i.e., statins or *n*-3 PUFAs) lowered the risks of total CVD (RelR): 0.89; 95% CI: 0.85, 0.94), CHD (RelR: 0.81; 95% CI: 0.75, 0.89), myocardial infarction (RelR: 0.78; 95% CI: 0.78, 0.85), and stroke (RelR: 0.91; 95% CI: 0.85, 0.98). Statins lowered the risks of total CVD, CHD, myocardial infarction, and stroke, with a RelR (95% CI) of 0.81 (0.76, 0.86), 0.70 (0.62, 0.77), 0.69 (0.61, 0.78), and 0.85 (0.79, 0.82), respectively. *N*-3 PUFAs decreased the risks of CHD (RelR: 0.81; 95% CI: 0.75, 0.89) and myocardial infarction (RelR: 0.89; 95% CI: 0.80, 0.99) in comparison with the control group. Statins had a significant LDL-C-lowering effect, which was not seen with *n*-3 PUFAs. This study suggests that statins, especially atorvastatin and pravastatin, may be more beneficial than *n*-3 PUFAs in reducing the risk of total CVD, CHD, and myocardial infarction.

Fan et al. pooled data from eight RCTs involving 803 patients to compare the effect of statins alone and statins combined with *n*-3 PUFAs on coronary artery plaques [123]. The combined treatment significantly decreased the progression of coronary plaque volume (SMD: −0.36; 95% CI: −0.64, −0.08; *p* = 0.01) and fiber content (SMD: −0.40; 95% CI: −0.68, −0.13; *p* = 0.004) compared with statin therapy alone. The subgroup analysis demonstrated that EPA therapy had a greater effect than the combination of EPA and DHA, but the difference was not statistically significant. Furthermore, the combined treatment decreased the plasma CRP level compared to statin monotherapy. There were no significant differences in plasma HDL-C or LDL-C or in lipid content in plaques between the two groups. These findings suggest that combined *n*-3 PUFA and statin treatment is superior to statin therapy alone in stabilizing and promoting coronary plaque regression and reducing the risk of further occurrence of cardiovascular events.

## 9. Summary and Conclusions

Management of ASCVD remains a challenge despite effective lipid-lowering treatments, as specified in current guidelines, since a residual cardiovascular risk persists [124]. While statins and *n*-3 PUFAs offer synergistic effects on dyslipidemia, the pleiotropic effects of the two overlap, including modulation of inflammatory markers and immune cell function, stabilization of atherosclerotic plaques, and improvement of endothelial function [19]. Furthermore, statins induce the endogenous synthesis of SPMs from AA and *n*-3 PUFAs, contributing to their inflammation-resolving properties. As *n*-3 PUFAs are major substrates for SPM synthesis, this may represent an important synergistic interaction between statins and *n*-3 PUFAs.

As chronic low-grade inflammation is involved in all stages of atherosclerotic diseases, inflammation is now considered an important therapeutic target [125]. Clinical evidence demonstrates that low plasma and tissue levels of SPMs are linked with the progression of ASCVD [85]. *n*-3 PUFAs, as SPM substrates promote their production [23], reduce inflammation [21,22], limit the progression of atherosclerotic plaques in animal models [126], and increase plaque stability [127,128]. Statin treatment may promote the production of SPMs, reducing inflammation and the progression of atherosclerosis. Studies on animal models show the potential therapeutic benefits of synthetic SPM analogs, either alone or in combination with statins, in attenuating atherosclerosis and promoting plaque regression [72]. These findings highlight the promising role of SPMs as novel therapeutic targets for ASCVD treatment [129].

The interaction between *n*-3 PUFAs and statins in CVDs has been investigated extensively through many long-term clinical trials and some recent meta-analyses, with the main focus being blood lipid and lipoprotein concentrations. In the leading RCTs, standard statins (moderate or high-intensity) were combined with relatively high doses solely of EPA or of EPA + DHA (up to 4 g/day), except for the JELIS trial, where subjects underwent low-intensity statin therapy. All studies (JELIS, REDUCE-IT, and EVAPORATE) using highly purified EPA (as icosapent ethyl) demonstrated cardiovascular benefits related to plaque reduction and stabilization and reduced coronary events. Despite having a similar study design and target populations, REDUCE-IT and STRENGTH had positive and null outcomes, respectively. As explained earlier, possible reasons for inconsistencies may relate to the dose of *n*-3 PUFAs used and the exact composition and formulation used. The ongoing RESPECT-EPA trial could elucidate whether highly purified EPA with current standard statins is effective in reducing cardiovascular events, considering the plasma ratio of EPA to AA as a biomarker of risk in CAD patients. Meta-analyses have provided additional insights into the efficacy of *n*-3 PUFAs and statins in reducing cardiovascular risk. These analyses have shown that combined *n*-3 PUFA and statin therapy is generally more effective than statin monotherapy in improving lipid profiles, stabilizing and promoting coronary plaque regression, and reducing the risk of further occurrence of cardiovascular events. This suggests that for optimal patient care, the combination of statins and high-dose *n*-3 PUFAs should be considered. Further research should further investigate SPMs as a target for combined therapy with statins and *n*-3 PUFAs.

## Figures and Tables

**Table 1:** Summary of different dosages (daily unless otherwise indicated) used for low-, moderate-, and high-intensity statin therapy.

Low-Intensity	Moderate-Intensity	High-Intensity
Simvastatin (10 mg)	Atorvastatin (10–20 mg)	Atorvastatin (40–80 mg)
Pravastatin (10–20 mg)	Rosuvastatin (5–10 mg)	Rosuvastatin (20–40 mg)
Lovastatin (20 mg)	Simvastatin (20–40 mg)	
Fluvastatin (20–40 mg)	Pravastatin (40–80 mg)	
Pitavastatin (1 mg)	Lovastatin (40 mg)	
	Fluvastatin XL (80 mg)	
	Fluvastatin (40 mg twice daily)	
	Pitavastatin (2–4 mg)	
